# Correlation of 4′-[methyl-^11^C]-thiothymidine PET with Gd-enhanced and FLAIR MRI in patients with newly diagnosed glioma

**DOI:** 10.1186/s13550-021-00785-8

**Published:** 2021-04-30

**Authors:** Takashi Norikane, Katsuya Mitamura, Yuka Yamamoto, Yukito Maeda, Kenichi Tanaka, Tetsuhiro Hatakeyama, Keisuke Miyake, Jun Toyohara, Yoshihiro Nishiyama

**Affiliations:** 1 Department of Radiology, Faculty of Medicine, Kagawa University, 1750-1 Ikenobe, Miki-cho, Kita-gun, Kagawa 761-0793 Japan; 2 Department of Clinical Radiology, Kagawa University Hospital, Kita-gun, Kagawa Japan; 3 Department of Neurological Surgery, Faculty of Medicine, Kagawa University, Kita-gun, Kagawa Japan; 4 Research Team for Neuroimaging, Tokyo Metropolitan Institute of Gerontology, Tokyo, Japan

**Keywords:** 4DST, PET, Glioma, MRI

## Abstract

**Purpose:**

To elucidate the biological association between tumor proliferation, tumor infiltration and neovascularization, we analyzed the association between volumetric information of 4′-[methyl-^11^C]thiothymidine (4DST) positron emission tomography (PET) and fluid-attenuated inversion recovery (FLAIR) and T1-weighted gadopentetate dimeglumine (Gd)-enhanced magnetic resonance imaging (MRI), in patients with newly diagnosed glioma.

**Methods:**

A total of 23 patients with newly diagnosed glioma who underwent both 4DST PET/CT and Gd-enhanced MRI before therapy were available for a retrospective analysis of prospectively collected data. The maximum standardized uptake value (SUVmax) for tumor (T) and the mean SUV for normal contralateral hemisphere (N) were calculated, and the tumor-to-normal (T/N) ratio was determined. Proliferative tumor volume (PTV) from 4DST PET and the volume of Gd enhancement (GdV) and hyperintense region on FLAIR (FLAIRV) from MRI were calculated.

**Results:**

All gliomas but 3 diffuse astrocytomas and one anaplastic astrocytoma had 4DST uptake and Gd enhancement on MRI. There was no significant difference between PTV and GdV although the exact edges of the tumor differed in each modality. The FLAIRV was significantly larger than PTV (*P* < 0.001). Significant correlations between PTV and GdV (*ρ* = 0.941, *P* < 0.001) and FLAIRV (*ρ* = 0.682, *P* < 0.001) were found.

**Conclusion:**

These preliminary results indicate that tumor proliferation assessed by 4DST PET is closely associated with tumor-induced neovascularization determined by Gd-enhanced MRI in patients with newly diagnosed glioma.

## Introduction

Tumor proliferation is considered an important indicator of tumor growth and therapeutic effectiveness [1]. Positron emission tomography (PET) using 2-deoxy-2-[^18^F]fluoro-D-glucose (FDG) is an established functional imaging modality for diagnostic oncologic imaging of various tumors [2]. However, since FDG directly reflects the glucose metabolism, it can provide only an indirect measure of cellular proliferation.

The most direct indicator of cellular proliferation is deoxyribonucleic acid (DNA) synthesis, which can be measured using radiolabeled thymidine or its analogs. A thymidine analog, 3′-deoxy-3′-[^18^F]-fluorothymidine (FLT), has been used to assess the tumor proliferative activity of various types of tumor including brain tumor [3–6]. In brain gliomas, FLT has been validated for evaluation of cellular proliferation and tumor grade [3]. Toyohara et al. developed 4′-[methyl-^11^C] thiothymidine (4DST) for cellular proliferation imaging, which is resistant to degradation by thymidine phosphorylase and is incorporated into DNA [7]. 4DST PET has been shown to be helpful in noninvasive evaluation of the proliferation of several types of tumor including brain tumor [8, 9].

Anatomical imaging using magnetic resonance imaging (MRI) has been the most commonly used modality for obtaining brain tumor information. Gadopentetate dimeglumine (Gd) enhancement in MRI is due to passive diffusion of the paramagnetic contrast agent across a damaged blood–brain barrier (BBB) and has been correlated with tumor neovascularization and proliferation [10]. Radiologically, glioblastoma usually manifests as a Gd-enhancing mass with surrounding non-enhancing tissue marked by abnormal fluid-attenuated inversion recovery (FLAIR) signal [11]. Pathology studies demonstrate microscopic tumor infiltration throughout the peritumoral edema, which appears hyperintense on FLAIR sequences [12]. More than 90% of tumor recurrences will occur within FLAIR envelope [12]. To elucidate the biological association between tumor proliferation, tumor infiltration and neovascularization, we analyzed the association between volumetric information of 4DST PET and FLAIR and T1-weighted Gd-enhanced MRI in patients with newly diagnosed glioma.

## Materials and methods

### Patients

We conducted a retrospective analysis of prospectively collected data. The prospective study consisted of 220 patients with brain tumors who underwent 4DST PET/CT study between August 2011 and July 2017. All patients included gave written informed consent, and the study protocol was approved by our institutional ethics committee. From these patients, 23 (9 males, 14 females; mean age, 59.1 years; age range, 21–84 years) were selected for this retrospective analysis. Eligible patients fulfilled all of the following criteria: (1) histologically confirmed glioma with treatment-naïve and (2) both 4DST PET/CT and T1-weighted Gd-enhanced and FLAIR MRI were performed within 2 weeks before treatment. The tumor numbers and types were as follows: 4 diffuse astrocytomas, 4 anaplastic astrocytomas, 1 anaplastic oligodendroglioma and 14 glioblastomas. This retrospective data collection was compliant with our institutional ethics committee, with the need for informed consent waived. Some of the data from 16 of these patients were used in a previous study [8].

### PET/CT and MRI studies

The 4DST was manufactured using an automated synthesis system with HM-18 cyclotron (QUPID; Sumitomo Heavy Industries Ltd, Tokyo, Japan) and was synthesized using the method described by Toyohara et al. [7]. All acquisitions were performed using a Biograph mCT 64-slice PET/CT scanner (Siemens Medical Solutions USA Inc., Knoxville, TN, USA). Data acquisition began with low-dose CT at the following settings: no contrast agent, 120 kV, 50 mA, 1.0-s tube rotation time, 3-mm slice thickness, 3-mm increments and pitch 0.55. PET emission scanning of the head region with a 15-min acquisition of one bed position was performed 15 min after intravenous injection of 4DST (6 MBq/kg). The PET data were acquired in 3D mode and were reconstructed with an ordered subsets expectation maximization, incorporating correction with point spread function and time-of-flight model (5 iterations, 21 subsets) on a 256 × 256 matrix, with a 21.6 cm axial field of view and 3 mm slice thickness.

MRI was performed using a 1.5 T MRI scanner (Achieva, Philips Medical Systems, Eindhoven, Netherlands). The MRI protocol for this study included the acquisition of 2D T2 FLAIR (repetition time/echo time = 8000/100 ms, 7 mm slice thickness with 1 mm interslice gap, matrix: 512 × 512, FOV 210 × 210 mm^2^) and 2D T1-weighted imaging (repetition time/echo time = 440/9 ms, 7 mm slice thickness with 1 mm interslice gap, matrix: 512 × 512, FOV 210 × 210 mm^2^) after administration of 0.1 mmol/kg Gd.

### Image analysis

PET images were visually assessed by two experienced nuclear physicians independently. Any difference of opinion was resolved by consensus. Tumor lesions were identified as areas of focally increased uptake, exceeding that of normal brain background. Semi-quantitative analysis using the standardized uptake value (SUV) was performed by an experienced nuclear physician using a syngo.via (Siemens Healthcare, Erlangen, Germany). The SUV was calculated using the following formula: SUV = *c*_dc_/(*d*_i_/*w*), where *c*_dc_ is the decay-corrected tracer tissue concentration (Bq/g); *d*_i_, the injected dose (Bq); and *w*, the patient’s body weight (g). The maximum SUV (SUVmax) for tumor and the mean SUV (SUVmean) for reference tissue were calculated. For the reference tissue, a circular region of interest (ROI) of 10 × 10 mm was manually placed on the uninvolved contralateral hemisphere. The tumor-to-contralateral normal brain tissue (T/N) ratio was determined by dividing the tumor SUVmax by the contralateral hemisphere SUVmean. Proliferative tumor volume (PTV) was defined as the volume with a threshold of 40% of the SUVmax [7]. When the radioactivity associated with the tumor could not be visually identified, the mean SUV of the tumor on the basis of MRI studies was measured and PTV was assumed to be zero.

In the MRI, ROI was manually placed over the Gd enhancing part of the tumor and the hyperintense region on FLAIR MRI. The volumes of the Gd enhancing part of the tumor (GdV) and the hyperintense FLAIR (FLAIRV) were calculated by summing the volumes (area multiplied by interslice distance) of the ROIs.

### Statistical analysis

Differences in semi-quantitative parameters were analyzed by nonparametric Wilcoxon signed-rank test. Spearman correlation coefficients were used to examine the relationship between PTV and GdV and FLAIRV. For all analyses, *P* values < 0.05 were considered statistically significant.

## Results

All gliomas but 3 diffuse astrocytomas and one anaplastic astrocytoma had 4DST uptake and Gd enhancement on MRI. The mean (± SD) of the normal brain parenchyma showed low 4DST uptake with an SUVmean of 0.40 ± 0.11. The mean (± SD) of the SUVmax for tumor and T/N ratio was 2.37 ± 1.65 and 5.82 ± 4.10, respectively.

**Fig. 1:**
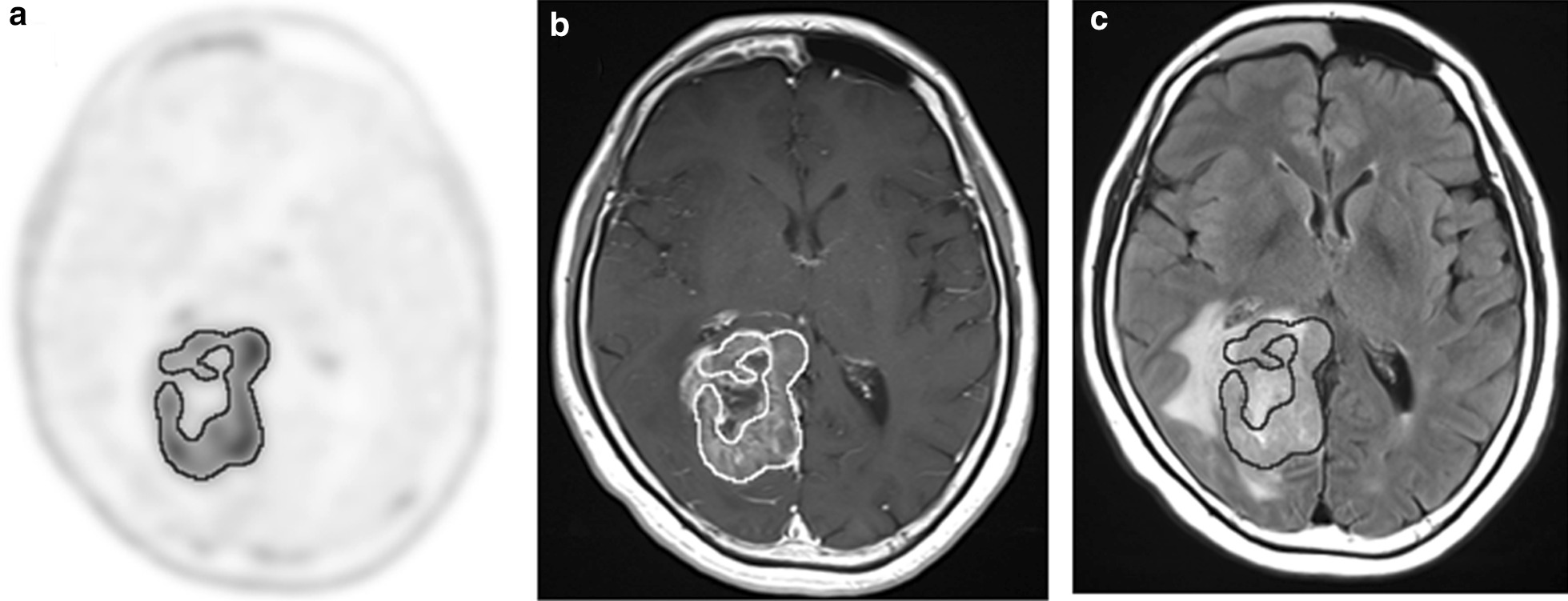
Radiologic images of a 60-year-old female with newly diagnosed glioblastoma. Transverse 4DST PET image **a** shows inhomogeneous uptake in the right occipital lobe (SUVmax = 5.36, T/N ratio = 13.74, PTV = 44.58 cm^3^). Transverse T1-weighted MRI with Gd enhancement **b** shows inhomogeneously enhanced lesion in tumor (GdV = 48.28 cm^3^). Region of interest shows proliferation area drawn on 4DST PET image from a. The proliferation area on 4DST PET image and Gd enhancement area on MRI differed very slightly. The hyperintense area identified on transverse FLAIR MRI **c** is larger than the proliferation area on 4DST PET image (FLAIRV = 137.52 cm^3^)

**Fig. 2:**
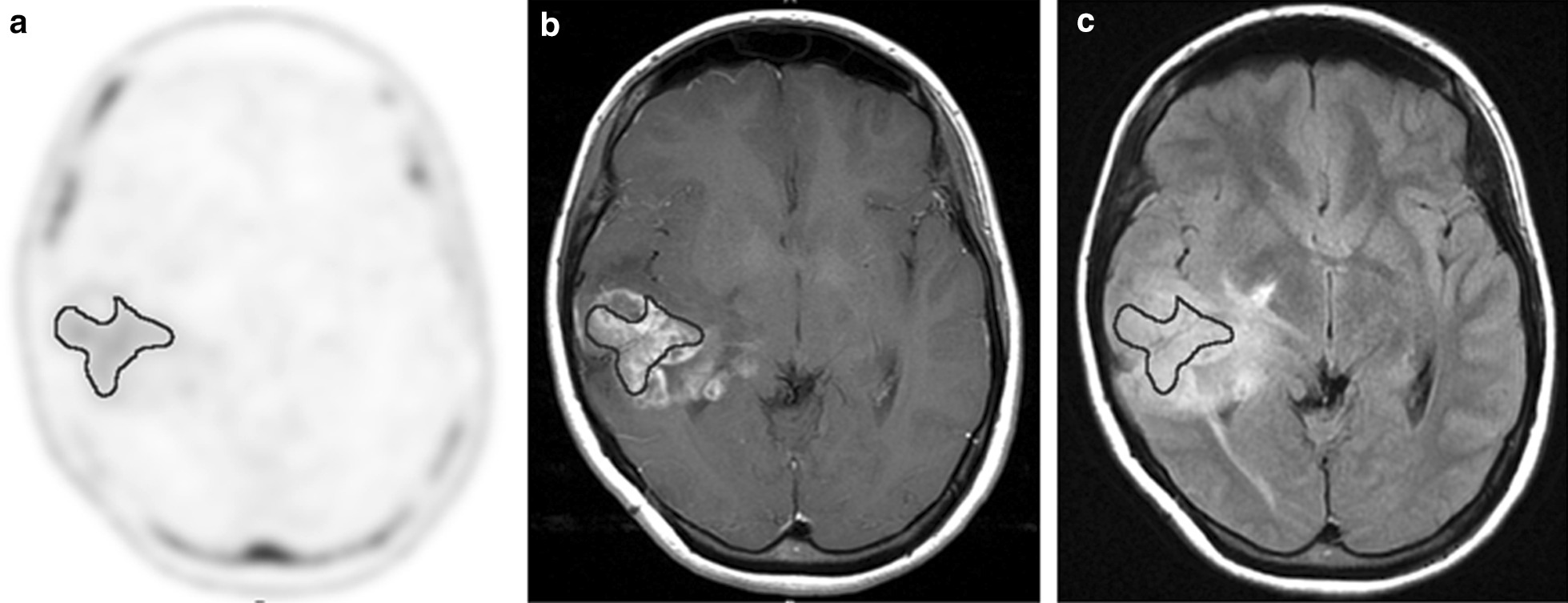
Radiologic images of a 29-year-old female with newly diagnosed glioblastoma. Transverse 4DST PET image **a** shows inhomogeneous uptake in the right temporal lobe (SUVmax = 1.87, T/N ratio = 5.84, PTV = 25.46 cm^3^). Transverse T1-weighted MRI with Gd enhancement **b** shows inhomogeneously enhanced lesion in tumor (GdV = 35.84 cm^3^). Region of interest shows proliferation area drawn on 4DST PET image from a. The proliferation area on 4DST PET image and Gd enhancement area on MRI differed slightly. The hyperintense area identified on transverse FLAIR MRI **c** is larger than the proliferation area on 4DST PET image (FLAIRV = 196.16 cm^3^)

**Fig. 3:**
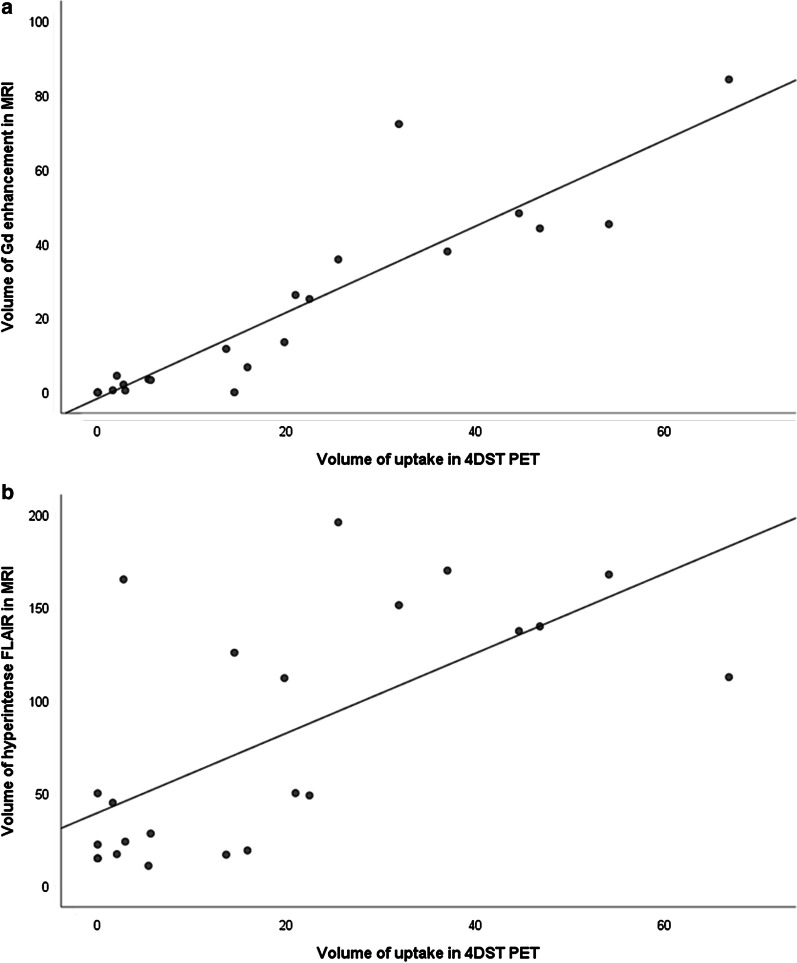
Plot of the volume of 4DST uptake and Gd enhancement in MRI (**a**), showing a significant correlation of the two parameters (Spearman correlation coefficient 0.941, *P* < 0.001). Plot of the volume of 4DST uptake and hyperintense FLAIR in MRI (**b**), showing a significant correlation of the two parameters (Spearman correlation coefficient 0.682, *P* < 0.001)

Table 1 shows the results of 4DST PET and MRI parameters in patients with newly diagnosed gliomas. In all patients, there was no significant difference between PTV and GdV although the exact edges of the tumor differed in each modality (Figs. 1, 2). The FLAIRV was significantly larger than PTV (*P* < 0.001). A significant correlation between PTV and GdV was found (*ρ* = 0.941, *P* < 0.001) (Fig. 3a). A significant correlation between PTV and FLAIRV was also found (*ρ* = 0.682, *P* < 0.001) (Fig. 3b).

**Table 1:** 4DST PET and MRI parameters in patients with newly diagnosed gliomas

Parameter	All patients (*n* = 23)	Glioblastoma (*n* = 14)
Median volume in cm^3^		
PTV (range)	14.47 (0–66.79)	23.94 (2.03–66.79)
GdV (range)	6.79 (0–84.35)	31.05 (3.35–84.35)
FLAIRV (range)	50.10 (11.02–196.16)*	112.45 (11.02–196.16)*

*PTV* Proliferative tumor volume, *GdV* volume of Gd enhancement, *FLAIRV* volume of hyperintense FLAIR

^*^P < 0.001 compared with PTV

In a subgroup analysis of patients with glioblastoma only, there was no significant difference between PTV and GdV. The FLAIRV was significantly larger than PTV (*P* < 0.001). Significant correlations between PTV and GdV (*ρ* = 0.921, *P* < 0.001) and FLAIRV (*ρ* = 0.754, *P* = 0.002) were also found.

## Discussion

We analyzed the association between volumetric information of 4DST PET and FLAIR and T1-weighted Gd-enhanced MRI in patients with newly diagnosed glioma. The results showed that the volume of 4DST uptake was closely correlated to the volume of Gd enhancement in MRI, but the exact edges of the tumor differed in each modality. These findings are similar to those from a previous study using FLT PET [4]. On the other hand, the FLAIRV was significantly larger than PTV.

FLAIR and Gd-enhanced MRI are commonly used to obtain anatomical information especially in the central nervous system. However, MRI provides nonspecific information of the tumor as it does not always differentiate brain tumors from non-tumorous tissue or low-grade tumors from high-grade ones. PET can provide valuable molecular information and may improve the ability of diagnostic procedures to determine the malignancy and the extent of tumor invasion.

Increased cellular proliferation and DNA replication are characteristic of malignant transformation [13]. The evaluation of cellular proliferation rate by means of PET is important as a noninvasive clinical approach. FLT, a fluorinated thymidine analogue, has emerged as a PET tracer for assessing tumor-proliferating activity in various tumors including brain gliomas [3–5]. However, little FLT is actually incorporated into DNA synthesis, and FLT uptake in the tumor does not reflect the whole DNA synthesis [14]. Due to the drawbacks of FLT, efforts were made to produce a more attractive PET tracer that can be used to accurately assess tumor malignancy and cellular proliferation. Toyohara et al. focused on the 4DST because of its metabolic stability and close similar structure to native thymidine [7].

After intravenous injection, 4DST distributes rapidly in the extracellular fluid and is transported from there into the cytosol by human nucleoside transporters, probably mainly by the human equilibrative nucleoside transporter-1 (hENT1) [15]. Plotnik et al. reported that proliferating cells have higher levels of hENT1 than non-proliferating cells [15]. 4DST is trapped within the cytosol after being monophosphorylated by thymidine kinase-1, one of the main enzymes in the salvage pathway of DNA synthesis [7, 15]. In the present study, the FLAIRV was significantly larger than PTV although there was no significant difference between GdV and PTV. The heterogeneity of glioblastoma is apparent on standard MRI sequences [12]. The peritumoral edema, which appears hyperintense on FLAIR sequence, appears to develop in response to angiogenic and vascular permeability factors associated with infiltrating tumor [12]. Gd enhancement in MRI is due to passive diffusion of the paramagnetic contrast agent across the damaged BBB and has been correlated with the tumor vascularization and proliferation [10]. In the present study, four gliomas without Gd enhancement could not be visualized with 4DST PET suggesting that 4DST does not easily cross the intact BBB. The present results showed that the volume of 4DST uptake was closely correlated to the volume of Gd enhancement in MRI although the exact edges of the tumor differed in each modality. Some areas showed 4DST uptake and no Gd enhancement, while other areas demonstrated Gd enhancement without 4DST uptake. The regional distribution of 4DST uptake with and without Gd enhancement in MRI suggests the presence of various tissue compartments in which 4DST uptake is due to the breakdown of the BBB, increased transport, increased proliferation or a variable combination of these components. 4DST PET may have a role not only in delineating neovascularization but also in evaluating the cellular proliferation and other aspects of tumorigenesis including invasion, progression and response to chemoradiation. The present study included only patients with newly diagnosed gliomas. The role of 4DST PET may be more evident in patients with post-treatment and recurrent gliomas in which MRI abnormalities were affected by radiation therapy. Further studies including histological analysis and patients with post-treatment and recurrent gliomas are needed to determine the relationship between 4DST uptake and Gd enhancement and FLAIR signal in the tumor.

Although 4DST PET is feasible for brain glioma imaging, its mechanism of uptake and biological significance are not yet understood. The value of the current data is limited by the retrospective character of the study and the small number of patients. The most important limitation was the lack of pathological correlation to the imaging findings, especially regarding the area of difference between 4DST uptake and Gd enhancement and FLAIR signal. Nowosielski et al. compared MRI with FLT and O-(2-[^18^F]fluoroethyl)-L-tyrosine (FET) PET in high-grade gliomas [16]. They concluded that FET but not FLT PET was able to detect metabolic active tumor tissue beyond contrast enhancing tumor on MRI [16]. To date, no reports have compared 4DST PET with FET PET in glioma. Accurate tumor detection and delineation before surgical resection are of great importance [17]. Nikaki et al. reported that the non-specific binding of FLT may be related to the false positive results, and could impair proper tumor delineation and characterization in gliomas [17]. The dynamic 4DST PET was not performed in the present study. A previous study by Toyohara et al. was performed brain dynamic 4DST scans and reported that physiologic uptake was observed in salivary glands, nasal mucosa and bone marrow [18].

Recently, Ceccon et al. evaluated the response to adjuvant temozolomide chemotherapy using FET PET and Gd-enhanced MRI in patients with newly diagnosed glioma [19]. They showed that relative changes of metabolic tumor volume (MTV) and T/N ratio obtained from FET PET, not MRI, significantly longer progression-free survival and overall survival (OS) [19]. Schwarzenberg and colleagues also reported that relative 3,4-dihydroxy-6-[^18^F]fluoro-L-phenylalanine (FDOPA) MTV change relative to baseline following bevacizumab and irinotecan predicted a significantly prolonged OS [20]. An important indication is the possibility that the cell proliferation imaging such as 4DST could be used for early treatment response assessment. There are currently no reports for treatment response assessment using 4DST PET in patients with gliomas. Further studies of larger numbers of patients are needed to address the clinical utility of 4DST PET for early evaluation of treatment response.

## Conclusion

These preliminary results in a small patient population indicate that tumor proliferation assessed by 4DST PET is closely associated with tumor-induced neovascularization determined by Gd-enhanced MRI in patients with newly diagnosed glioma. Further prospective studies, with greater number of patients, will demonstrate the usefulness of 4DST PET for tumor delineation, treatment response and prognosis, in patients with glioma.

## Data Availability

All results are provided in the manuscript.
